# Black pepper and tarragon essential oils suppress the lipolytic potential and the type II secretion system of *P. psychrophila* KM02

**DOI:** 10.1038/s41598-022-09311-9

**Published:** 2022-03-31

**Authors:** Natalia Tomaś, Kamila Myszka, Łukasz Wolko

**Affiliations:** 1 Department of Biotechnology and Food Microbiology, Poznan University of Life Sciences, Wojska Polskiego 48, 60-637 Poznań, Poland; 2 Department of Biochemistry and Biotechnology, Poznan University of Life Sciences, Dojazd 11, 60-632 Poznań, Poland

**Keywords:** Food microbiology, DNA sequencing, Antimicrobials

## Abstract

Given the increasing consumer demand for raw, nonprocessed, safe, and long shelf-life fish and seafood products, research concerning the application of natural antimicrobials as alternatives to preservatives is of great interest. The aim of the following paper was to evaluate the effect of essential oils (EOs) from black pepper (BPEO) and tarragon (TEO), and their bioactive compounds: limonene (LIM), β-caryophyllene (CAR), methyl eugenol (ME), and β-phellandrene (PHE) on the lipolytic activity and type II secretion system (T2SS) of *Pseudomonas psychrophila* KM02 (KM02) fish isolates grown in vitro and in fish model conditions. Spectrophotometric analysis with the p-NPP reagent showed inhibition of lipolysis from 11 to 46%. These results were confirmed by RT-qPCR, as the expression levels of *lipA*, *lipB,* and genes encoding T2SS were also considerably decreased. The supplementation of marinade with BPEO and TEO contributed to KM02 growth inhibition during vacuum packaging of salmon fillets relative to control samples. Whole-genome sequencing (WGS) provided insight into the spoilage potential of KM02, proving its importance as a spoilage microorganism whose metabolic activity should be inhibited to maintain the quality and safety of fresh fish in the food market.

## Introduction

Fresh fish and minimally processed fish-based products are susceptible to spoilage caused by microbiological reactions, which results in large economic losses for the fish industry and leads to sensory impairment^1,2^. It was stated that one-fourth of the world’s food supplies and 30% of landed fish/fish-based products are lost through microbial activity alone^3^. Lipid deterioration can easily take place limiting the shelf-life of aerobically stored fishery products^4^.

*Pseudomonas* spp. contribute to seafood spoilage by synthesizing extracellular lipases that hydrolyze triglycerides into free fatty acids, mono- and diacylglycerols, and glycerol, which undergo further degradation to create off-flavor low-molecular weight compounds. Lipases are usually very heat stable^5^. Even a small degree of lipolysis (1–2%) of fat can change the taste of products^6^. *Pseudomonas psychrophila* has emerged as the dominant fish-associated pseudomonads and possess the strongest potential to synthesize lipases secreted via the type II secretory pathway (T2SS)^1,7,8^. Regulation of this bacterial activity involves not only the level of lipase gene transcription and the translation of a particular mRNA but also subsequent translocation through the cell wall^9^. Because treatment that inactivate the T2SS results in loss of secretion of enzymes, combining that treatment with one that downregulate the expression of genes encoding lipases is considered a target for food quality improvement interventions^10^. However this concept requires further analysis involving integrated molecular and phenotype-based approaches. Supplementing studies with a whole genome sequencing (WGS) and de novo assembly data can provide insight into the functional profile/food spoilage features of *P. psychrophila*. WGS data can allow for characterization of potential hazards to the quality and safety of foods associated with microorganisms^11^. Importantly, complete genome assembly which is superior to the study of genome fragments and provides the only accurate reference for interpreting the meta-genomes and -transcriptomes^12^ of this species has not been performed to date.

The work of Steniša et al.^13^ indicates that the presence and metabolic activity of *P. psychrophila* in seafoods must be controlled. In general, growing concerns regarding the use of synthetic preservatives associated with the selection of resistant strains and the triggering of allergic reactions in consumers has prompted research into the development of new solutions to reduce microbial load and microbe activities in fishery products. Recently, interest into the use of essential oils (EOs) in seafoods has been growing. Black pepper EO (BPEO) and tarragon EO (TEO) are EOs that may prolong shelf life while maintaining the fresh characteristics of fish^8,14^. In the current study, we used limonene (LIM) (19.1%) and β-caryophyllene (CAR) (19.6%)-rich BPEO and TEO where methyl eugenol (ME) (24.5%) and β-phellandrene (PHE) (19.3%) were the most abundant compounds. The chemical profiles of BPEO and TEO determined by gas chromatography coupled to mass spectrometry were presented in our previous works^8,14^. The contents of the oils correspond with the international standard regulations that set minimum and maximum concentrations of LIM and CAR for BPEO and ME and PHE for TEO^15,16^.

BPEO and TEO extended the shelf life of chill stored carp^17^, talang gueenfish^18^, and brook trout^19^. The mode of activity of the oils affects bacterial membrane structures, which changes the permeability of the bacterial cell wall. This can be accompanied by disruption in membrane function, such as electron transfer and enzyme activities^20^. The interference of the bioactive compounds of the oils with nucleic acids was also recognized as a possible antimicrobial activity of EOs^21^. However, the influence of BPEO and TEO on the expression of genes involved in lipase synthesis and secretion in the presence of the food spoiler *P. psychrophila* has not been evaluated. The above information can support the development of new technologies to prevent lipids deterioration in seafoods.

In this work, the inhibition of lipolysis of *P. psychrophila* KM02 (KM02) by BPEO, and TEO was investigated. KM02 cells were incubated with subinhibitory concentrations (subMICs) of the analyzed agents in vitro and in fish juice medium and the following aspects were examined: (i) the changes in lipolytic activity by a spectrophotometric method with p-NPP reagent, (ii) the expression of genes encoding lipases and T2SS by RT-qPCR analyses, and (iii) the growth inhibitory activity during vacuum packaging of marinated salmon fillets supplemented with BPEO and TEO. Additionally, to better understand and investigate the metabolic potential of the KM02 isolate, this work was introduced with a characterization of the whole genome, sequenced by Illumina and Nanopore techniques and de novo assembly.

## Materials and methods

### Microorganism and culture conditions

The KM02 strain isolated from commercial chill-stored fresh salmon was used in this study. Identification of restriction length polymorphisms of 16S rRNA gene amplicons and sequencing were carried out for bacterial identification. Cryovials (MWE, UK) were used for the preservation the strain.

The cells were cultured in tryptic soy broth medium (TSB) (BD Biosciences, USA) and fish juice medium prepared from fresh salmon fillets for a total of 72 h at 4 °C as described in the work of Sobieszczańska et al.^8^ The media were supplemented with previously selected^8,14^ subMICs of BPEO (135.0 µl/ml), LIM (Sigma–Aldrich, Merck KGaA, USA) (65.0 µl/ml) CAR (Sigma–Aldrich, Merck KGaA, USA) (35.0 µl/ml), TEO (75.0 µl/ml), ME (Sigma–Aldrich, Merck KGaA, USA) (10.0 µl/ml), and PHE (Sigma–Aldrich, Merck KGaA, USA) (8.0 µ/ml). The subMICs of the examined agents were determined by the macrodilution method, following the standard protocol M07 from the Clinical and Laboratory Standard Institute^22^.

### Whole genome analysis of *P. psychrophila* KM02

KM02, grown on TSB (Becton Dickinson, USA) medium, was subjected to whole genome sequencing analysis at a commercial laboratory (Genomed S.A., Poland). The bacterial genomic DNA was extracted with a Qiagen DNeasy Blood and Tissue kit (Qiagen, Germany) according to the manufacturer’s instructions, followed by fragmentation by sonication. Genome sequence and library preparations were constructed by two methods: the Illumina MiSeq Platform (Illumina, USA) and MinION sequencer (Oxford Nanopore Technologies, UK) by using the Nextera XT DNA library 300-bp paired-end preparation kit and the SQK-NSK007 Rapid Sequencing kit, respectively.

Raw sequencing data were processed with the CLC Genomics Workbench v. 20.0 and CLC Microbial Genomics Module v 20 plugin (Qiagen, USA). The sequence reads from the MiSeq Illumina platform were demultiplexed to the probes, and the overlapping paired-end reads were merged. Only fragments that passed the merging (~ 90%) were retained for downstream processing. Then, the reads from both the MiSeq and MinION sequencing platforms were combined together and used for de novo assembly. Sequence assembly was conducted using an increasing word size (k = 21, 41, 61), where the contigs from the previous iteration were used as input in the next iteration together with the input reads. The complete sequence of the bacterial chromosome was deposited in the NCBI GenBank database under accession number NZ_CP049044.1. Whole genome alignments were performed using Mauve 2.4.0^23^. Genome visualization was created with the GView Server (https://server.gview.ca)^24^. Pangenome analysis with KEGG and COG distributions was calculated using BPGA 1.3^25–28^. The phylogenetic tree was constructed based on genome assembly sequences by the K-mer method, with the following parameters: K-mer length = 16, index k-mers with prefix ATGAC, and method FFP.

Protein coding sequences (CDSs) were assigned to the KM02 whole genome sequence using the Find Prokaryotic Genes tool in the CLC Microbial Genomics Module. Thereafter, functional annotations of CDSs were performed using SwissPROT (with Gene Ontology (GO)-term annotations) and the protein family (Pfam) database. In the next step, the original reads were mapped back to the annotated genomes to assess the abundance of the functional annotations and build functional profiles. The COG distributions of KM02 were calculated using the WebMGA internet service (http://weizhong-lab.ucsd.edu/webMGA/server/)^29^.

### Lipolytic activity determination

The changes in KM02 lipolytic activity after treatment with subMICs of BPEO, TEO, and their major bioactive compounds were estimated by spectrophotometric analysis^30^. Briefly, 10 ml isopropanol containing 30 mg of p-nitrophenyl palmitate (p-NPP) (Sigma–Aldrich, Merck KGaA, USA) was mixed with 90 ml of 0.05 M Sörensen phosphate buffer (Sigma–Aldrich, Merck KGaA, USA). First, 2.4 mL was prewarmed at 37 °C and then mixed with the supernatant of the KM02 culture (0.1 mL). The samples were then incubated at 37 °C for 15 min. The absorbance was read at 420 nm on a SPECORD®UV–VIS spectrophotometer (Analytic Jena, Germany). Samples mixed with 0.1 mL of water served as reference probes. The percentage of lipolytic activity inhibition was calculated according to the following formula:

$$ \% {\text{ LI }} = { 1}00 \, {-} \, \left( {{\text{A}}_{{\text{t}}} /{\text{A}}_{{\text{c}}} *{ 1}00} \right) $$

where A_t_—absorbance value obtained for KM02 incubated on medium supplemented with subMICs. A_c_—absorbance value obtained for the control probe (KM02 cells without any treatment).

### RNA isolation and RT-qPCR analyses

The comparative quantitation method for evaluating the changes in the expression of the selected genes (Supplementary Table S1) was performed as described in our previous study with some modifications^14^. Briefly, total RNA was stabilized by RNAprotect® Bacteria Reagent (Qiagen, USA) and isolated on a PureLink™ RNA Mini Kit (Thermo Fisher Scientific, USA) followed by purification with PureLink™ DNase Set (Invitrogen, USA) according to the manufacturer's protocols. The quality and quantity of RNA were examined on a Qubit Fluorometer 4 (Invitrogen, USA) using Qubit™ XR RNA and Qubit™ IQ RNA Assay Kits (Thermo Fisher Scientific, USA). Subsequently, 1 µg of RNA was reverse-transcribed with the High Capacity RNA-to-cDNA Kit (Life Technologies, USA).

RT-qPCR analyses were performed in a CFX96 system (BioRad, Hercules, USA) using GoTaq® Master Mix (Promega, Germany). In the RT-qPCR analyses, 16S rRNA served as the reference gene. For the selected genes of interest (GOIs) the primers were designed with the Primer-BLAST tool^31^ in the NCBI database. The cycling conditions were as follows: initial denaturation at 95 °C for 2 min and 40 cycles of denaturation at 95 °C for 15 s and annealing and extension at 60 °C for 1 min. The melting curve was also applied. To estimate the amplification efficiency (*E*_GOI_/*E*_*ref*_), LinRegPCR software^32^ was used. The results are presented as the ratio of gene expression in the treated samples relative to the control samples (with expression equal to 1), normalized to the internal reference gene, according to the following equation^33^:

$$ratio= \frac{{{E}_{GOI}}^{{\Delta C}_{t target} (control-sample)}}{{{E}_{ref.}}^{{\Delta C}_{t ref} (control-sample)}}$$

where GOI—gene of interest for which the changes in expression were calculated; ref—reference gene, whose expression was used for normalization.

### In situ determination of the antimicrobial properties of BPEO and TEO toward *P. psychrophila* KM02

Ten grams of commercial raw salmon fillet samples were inoculated with 1 mL of KM02 culture standardized to an initial level of approximately 10^4^ CFU/g. The samples were air-dried at room temperature for 20 min in a biosafety laminar box (Thermo Fisher Scientific, USA). The marinade of the salmon consisted of 95% olive oil and 5% vinegar. All ingredients were purchase from local manufacturers. Next, 5 mL of marinade was supplemented with subMICs of BPEO/TEO. Marinade without essential oils served as reference samples. The marinades were poured into the salmon samples. The fish were packed into sterile polyvinyl chloride bags (Kraina Foils Packaging, Poland). Vacuum conditions were obtained with a Multivac T200 packaging machine (Wolfertschwenden, Germany). All samples were prepared under sterile conditions and stored at 4 °C ± 1 °C for 5 days.

Verification of KM02 growth on the cold-stored products was carried out at 1, 3 and 5 days of storage. The products were aseptically opened and placed in a sterile polyethylene bag (Sigma-Aldrich, Merck, KGaA, USA). A volume of 90 mL of 0.1% sterile peptone water (Oxoid, UK) was added to achieve a 1:10 dilution. The samples were homogenized for 2 min with a Pulsifier (Microgen Bioproducts, UK). Next tenfold serial dilutions were prepared, and 0.1 mL aliquots were surface-spread on cephalordine fucidin cetrimide agar (Oxoid, UK). Counts of KM02 are presented as the log CFU/g value.

### Statistical analysis

The experiments were performed in triplicate, and the results are expressed as the mean ± standard deviation. Significant differences (p < 0.05) were established by analysis of variance (ANOVA) followed by post hoc tests performed in R (R Core Team 2020).

## Results and discussion

### Characteristics of the *P. psychrophila* KM02 genome and pangenome

The growth and metabolic activities of microorganisms are considered the main causes of fish-based product spoilage. Our previous work identified *P. psychrophila* as a specific spoilage organism in the salmon microbiome^8^. At temperatures that can occur during the processing and storage of fish, the enzymatic activity of pseudomonads can result in off-odors^34^. However, determination of the physiological characteristics of *P. psychrophila* in in vitro and in situ conditions requires detailed investigation. WGS and de novo assembly provide complete information about bacterial strains isolated from various sources and increase the value of biological studies^12^. These are important because foodborne microorganisms are subjected to harsh conditions in the food chain^35^*.* Therefore, in this study, WGS technology was applied to understand the spoilage potential of the KM02 strain.

**Figure 1:**
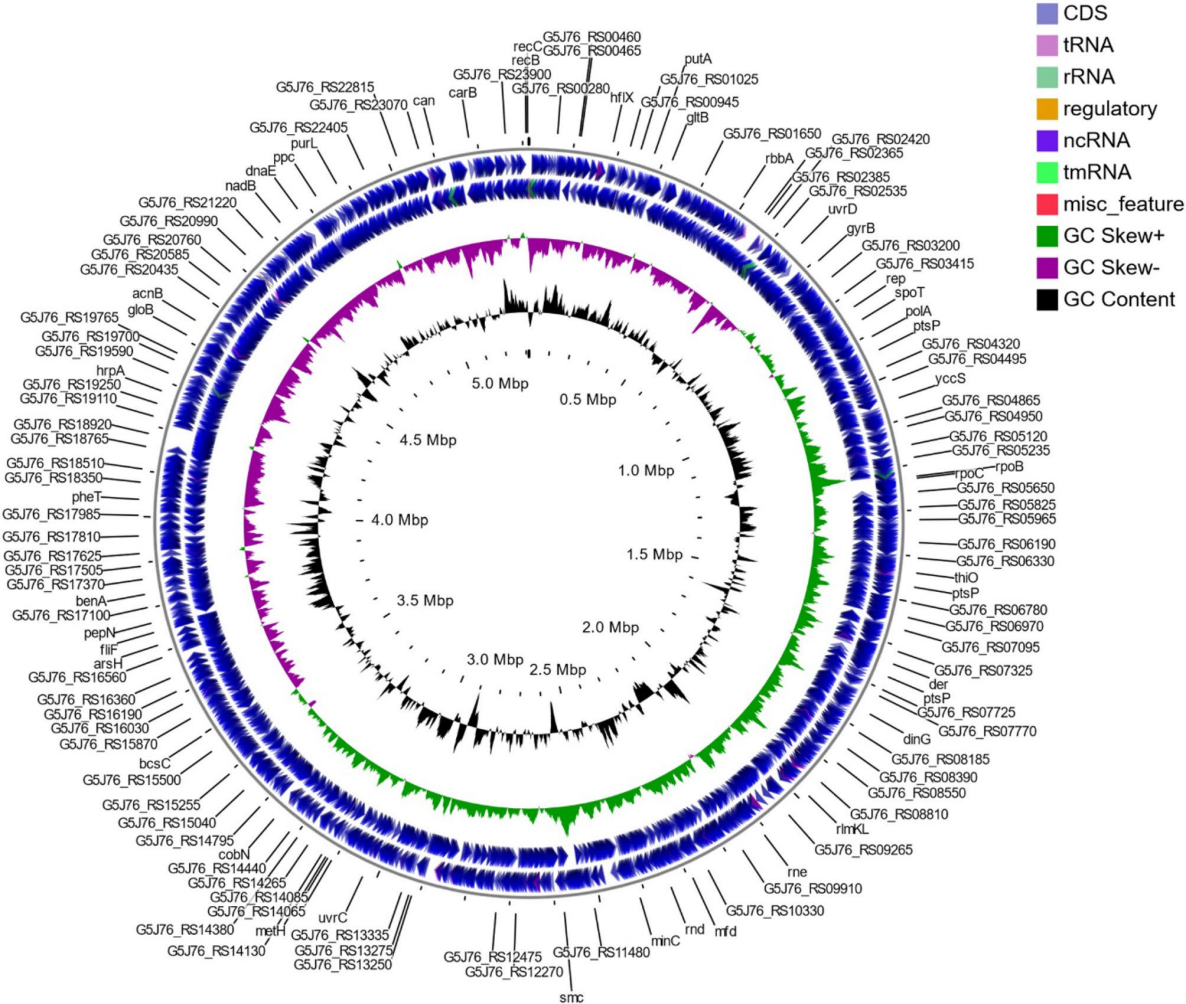
Circular representation of the *P. psychrophila* KM02 genome.

Initially, KM02 was isolated from salmon fillets kept in a cold environment, and the complete genome sequence was obtained by combining the Ilumina and MinION platforms. Reads were de novo assembled, resulting in one scaffold with 20 × coverage. A complete genome sequence of KM02 comprised 5,313,922 base pairs of a circular chromosome with a 57.4% G + C content (Fig. 1), coding 4,813 total genes, out of which 4,713 were protein coding DNA sequences (CDSs) and 54 were pseudogenes. No plasmids were found. Other chromosome features of KM02 are presented in Supplementary Table S2. In comparison to KM02, a previously sequenced genome of the *P. psychrophila* HA-4 strain also isolated from a cold environment, consisted of 5,235,696 bases with a mean G + C percentage of 56.4% and 4,721 predicted coding sequences^36^. Similarly, the draft genome of *P. psychrophila* MTCC 12,324 isolated from the Arctic was composed of 5,269,174 bases, with a mean G + C content of 57.52%^37^. Given the above, it can be concluded that *P. psychrophila* species genome characteristics are rather equal between strains, in contrast to other species, such as *Escherichia coli*^38^. However, only small variations in *P. psychrophila* genome features were able to be identified by analyzing the small number of available sequenced strains; many more sequences strains are available for *E. coli* species, and identification of genome variations is dependent on the number of genomes available for analysis^38^. Nevertheless, according to the work of Wessels et al.^39^, who sequenced 35 various *Pseudomonas* spp. fish- isolates, the genome sizes and number of genes ranged from 4,505,98 bp to 6,279,60 bp and 4,123 to 5,874, respectively^39^. Similar values were also obtained for genomes of *Pseudomonas fragi* and *Pseudomonas lundensis* isolated from spoiled meat and milk samples^40^. Based on the summary of the available sequenced bacterial genomes, such values are considered average, characteristic for bacteria isolated from environmental sources^38^.

**Figure 2:**
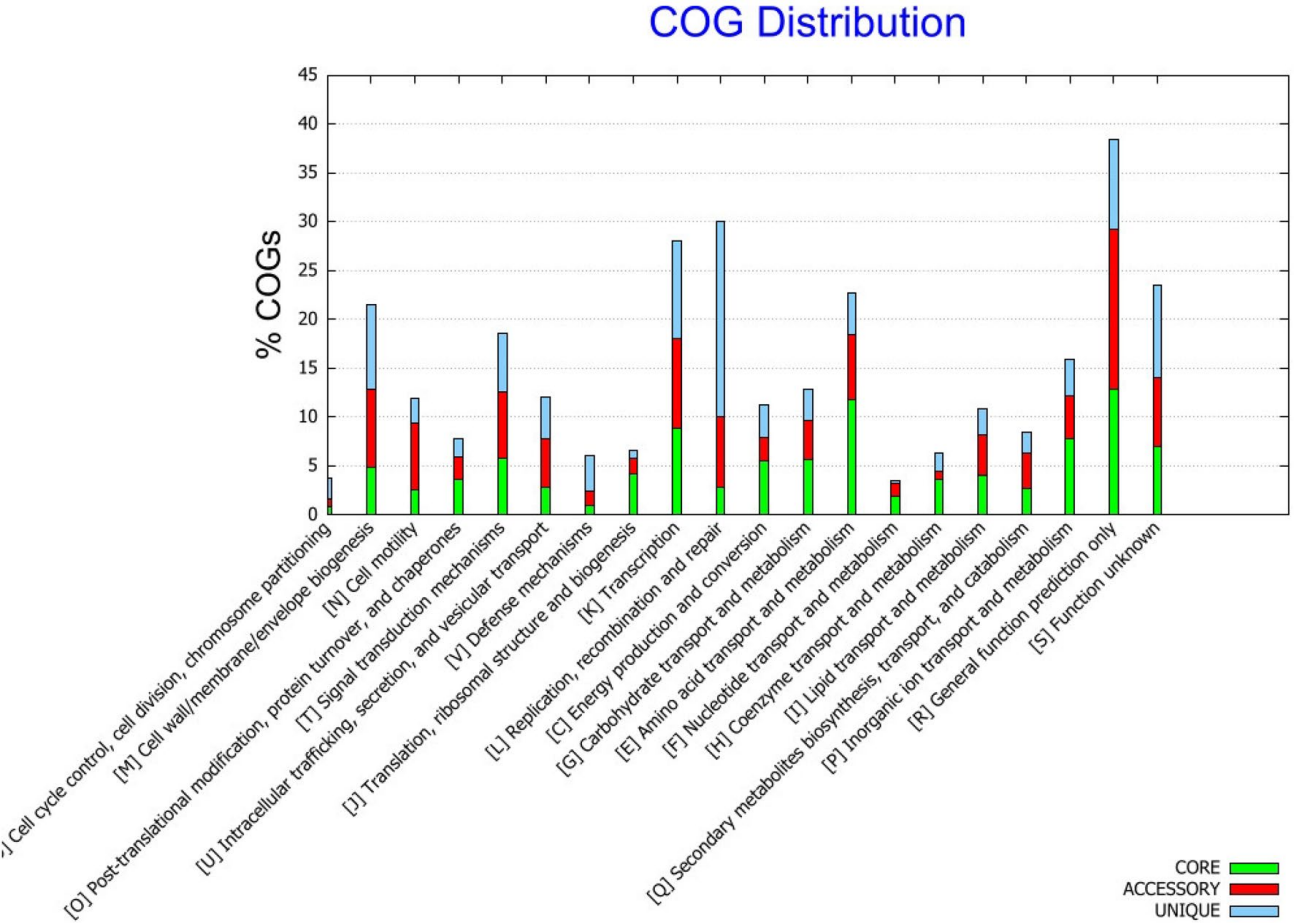
Clusters of orthologous groups distribution of *P. psychrophila* pangenome.

**Figure 3:**
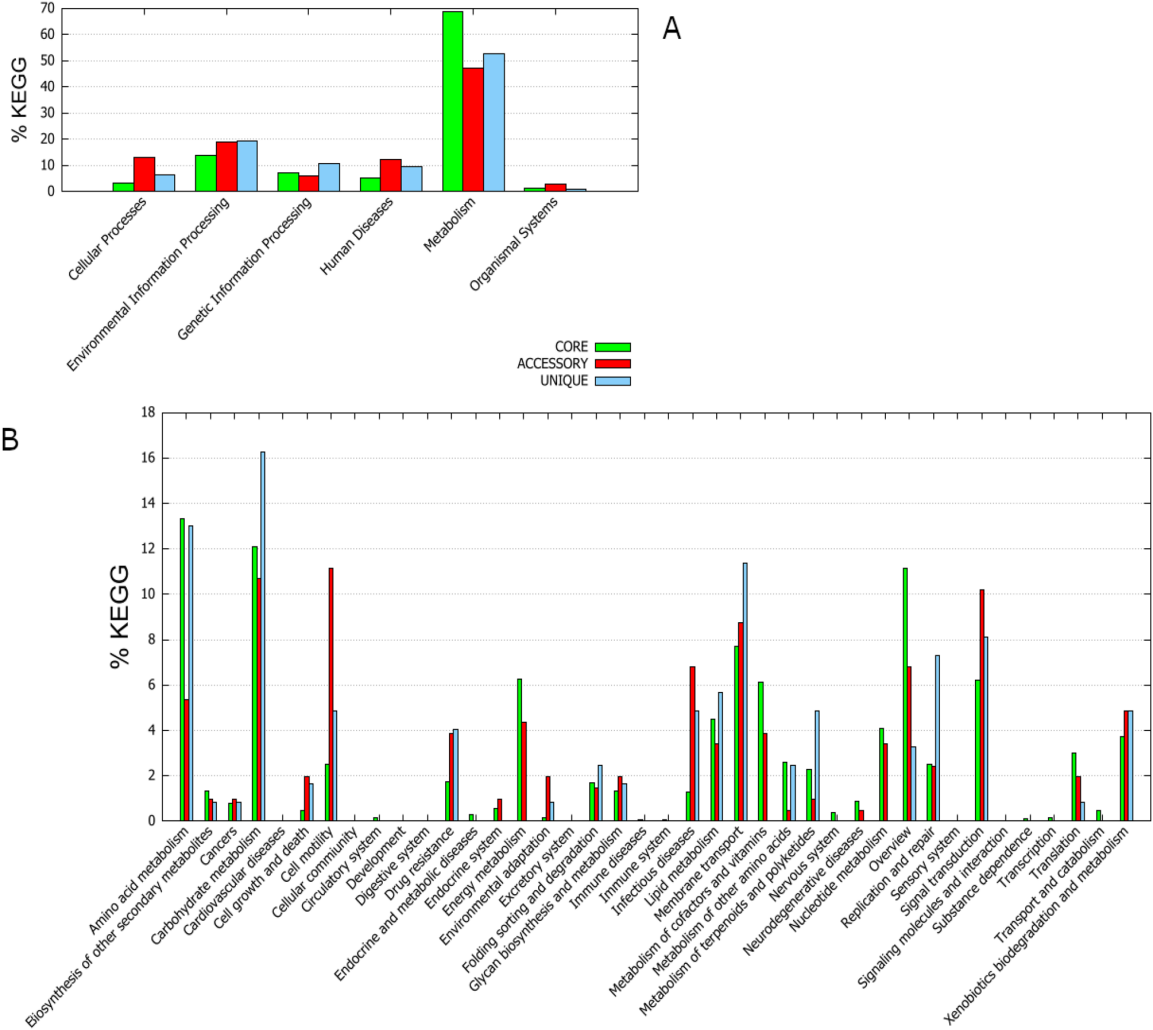
KEGG distribution of *P. psychrophila* pangenome. (**A**) General distribution; (**B**) details distribution.

To assess the essential genomic elements of *P. psychrophila* species, pangenome analysis was performed. There were 8 *P. psychrophila* genome assemblies in the GenBank NCBI database. Due to problems with gbff file validation HA-4 assembly (GCA_000282975.1) was excluded from the pangenome analysis. Details on the contribution of specific *P. psychrophila* genomes to the pangenome of this species are depicted in Table 1. *P. psychrophila* was characterized by 3914 core genes (shared by all strains), while strain KM02 had 548 accessory genes (shared by two or more strains, but not all), 2 unique and 0 absent genes. In the most distinct strain, MF6762, the number of unique genes reached 574. Interestingly, this strain has also been isolated from food (raw chicken), while other sequenced *P. psychrophila* strains were isolated from cold environments (rooms of food storage or cold water). As presented in the core-pangenome plot (Supplementary Fig. S2), *P. psychrophila* had a small variations among strains and varied only in the range of less than 2 Mb of gene families. This indicates a rather confined and homogeneous group of *P. psychrophila* strains in the context of gene products^38^. According to the outcomes of the attribution of the Clusters of Orthologous Groups (COGs) functional categories to the *P. psychrophila* pangenome (Fig. 2), the highest percentage of unique genes was related to the ‘replication, recombination and repair’ category. Simultaneously, the fraction of pangenome core, after the ‘genes of general function prediction only’ category, was the most abundantly represented in ‘amino acid transport and metabolism’ (above 10%). Similar results were obtained for the pangenome of fish-pathogenic *Aeromonas hydrophila* strains, in which the core genome was represented by 9.61% of genes annotated to the ‘amino acid transport and metabolism function’ COG category^41^. This functional group was also well represented in the accessory genome of *P. fragi*, which has been recognized as a contributor to the spoilage of fresh meat and fish and pasteurized milk by secreted lipases and proteases^40^. Regarding lipid transport and metabolism, the core, accessory and unique percentages of representatives in *P. psychrophila* pangenome were equally distributed. Furthermore, based on KEGG pangenome analysis (Fig. 3), a high metabolic activity of *P. psychrophila* (almost 70% of the pangenome) was also confirmed. KEGG detailed distribution revealed that the most enriched metabolic functions were ‘amino acid metabolism’, ‘lipid metabolism’ and ‘membrane transport’ and they accounted for approx. 13%, 4%, and 8% of the core genes, respectively.

**Table 1:** *P. psychrophila* pangenome characteristics.

Strain	Assembly level	Isolation source	Total sequence length	GenBank assembly accession	No. of core genes	No. of accessory genes	No. of unique genes	No. of exclusively absent genes
KM02	Complete Genome	Raw salmon fillets kept in cold	5,313,922	GCA_011040435.1	3,914	548	2	0
BS3667	Chromosome	Unknown	5,322,478	GCA_900106105.1	3,914	554	42	13
DSM 17,535	126 contigs	Cold room for food storage	5,334,010	GCF_001043005.1	3,914	489	41	14
CCUG 53,877	36 contigs	Cold room for food storage	5,269,270	GCA_008801485.1	3,914	537	26	2
MF6762	77 contigs	Raw chicken	5,804,172	GCF_016405605.1	3,914	427	574	27
CF149	50 contigs	Hyporheic zone of the Clark Fork River	5,154,320	GCA_000416155.1	3,914	382	219	48
RGCB 166	150 contigs	Surface water of the Arctic Fjord	5,269,174	GCA_001005765.1	3,914	263	144	133
HA-4	145 contigs	Activated sludge sample	5,235,696	GCA_000282975.1	–	–	–	–

**Figure 4:**
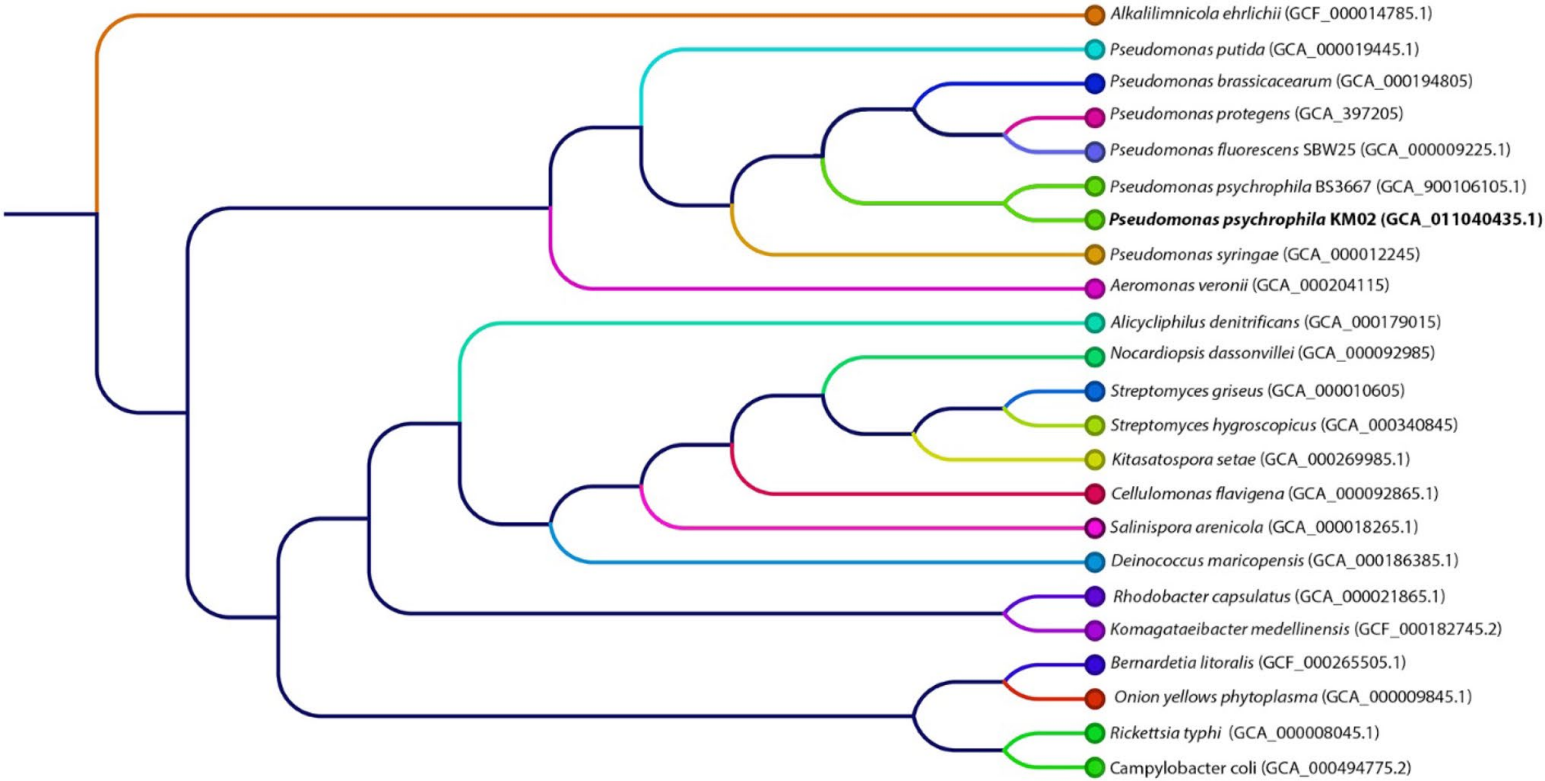
K-mer phylogenic tree of *P. psychrophila* KM02.

A K-mer phylogenetic tree based on the genome assembly sequences showed the closest evolutionary relationship with *P. psychrophila* BS3667 (Fig. 4) with 99.9181% identity; however the sample and isolation source of this strain is not known.

**Figure 5:**
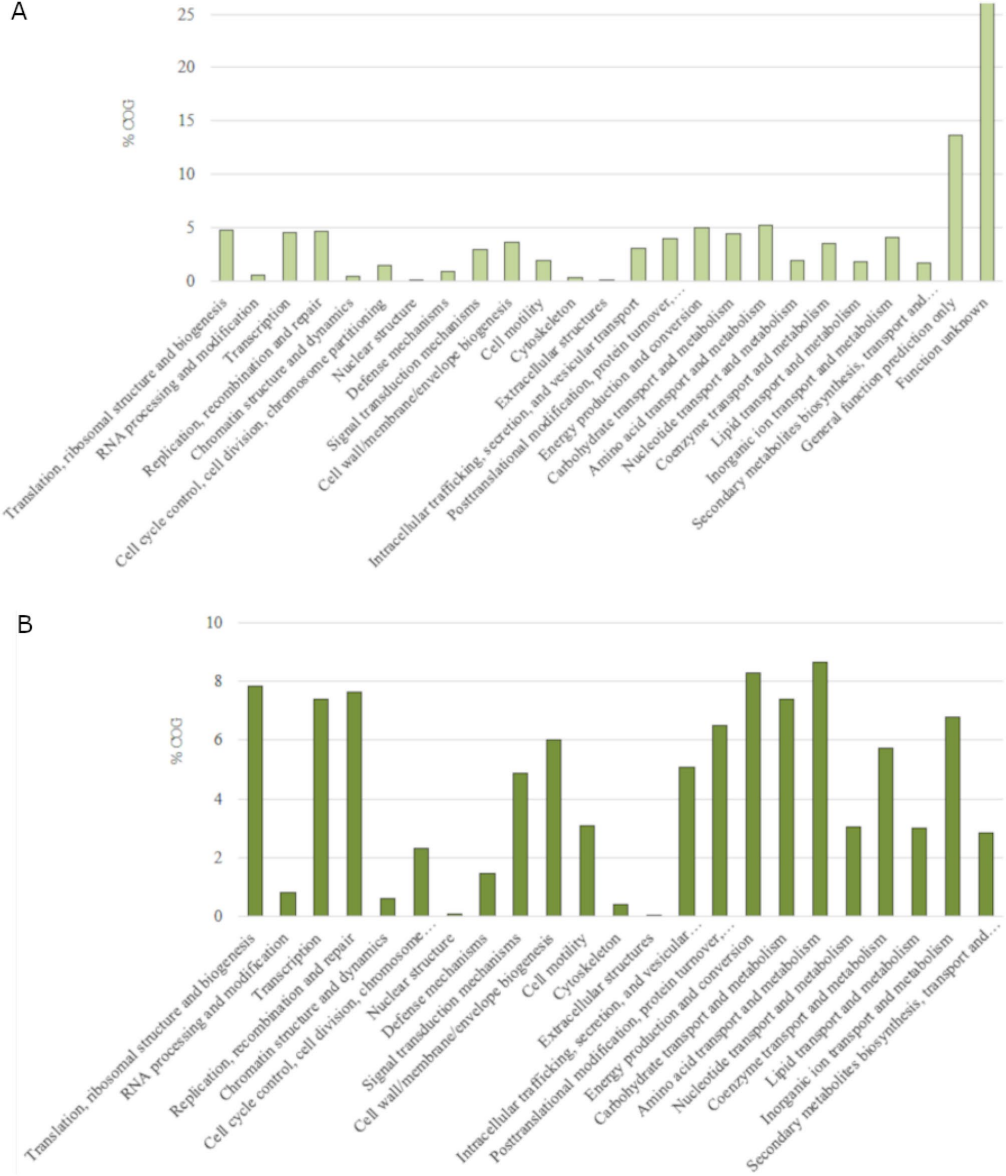
COG distribution of *P. psychrophila* KM02 genome.

**Figure 6:**
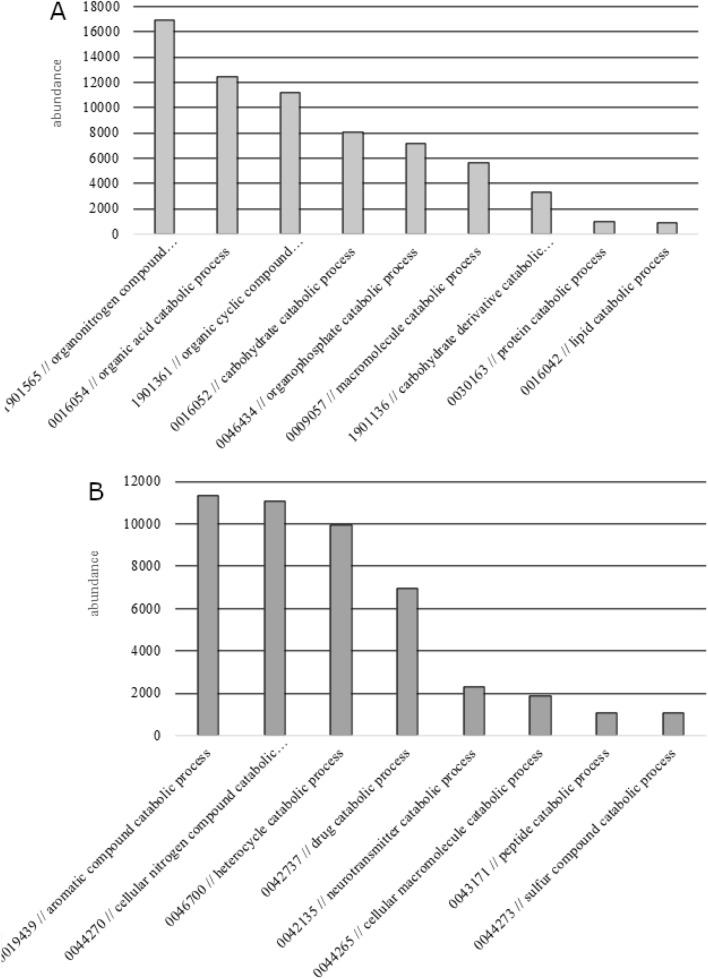
Gene abundance of selected GO terms in *P. psychrophila* KM02 genome. (**A**) Selected child terms of ‘organic substance catabolic process’ GO term. (**B**) Selected child terms of ‘cellular catabolic process’ GO term.

To characterize the KM02 genome, a number of available bioinformatic tools were applied. Similar to pangenome analysis, the distribution of COG of the KM02 strain was also determined. As presented in Fig. 5, gene products with ‘unknown function’ and with ‘general function prediction only’ comprised approx. 40% in total. Therefore, to clarify the significance of the remaining known function assigned to the COG, the numbers of those families were subtracted from the percentage calculations. Consequently, ‘amino acid and lipid transport and metabolism protein’ orthologs accounted for 9 and 3% of all categorized proteins, respectively. COG results for KM02 are in agreement with the genome of other seafood spoilage contributors, such as *Shewanella baltica* isolated from spoiled shrimp^42^. These psychrotrophic bacteria, similar to *Pseudomonas* spp. dominate in spoilage of iced-stored fish meat^43,44^ and the COG category of ‘amino acid’ and ‘lipid transport and metabolism’ represented 8.66% and 3.8% of the genome, respectively^42^. In regard to the proteins related to those activities, the most dominant annotations involved COG1028 (dehydrogenases with different specificities), COG0834 (ABC-type amino acid transport/signal transduction system), COG0665 (glycine/D-amino acid oxidases), COG1280 (putative threonine efflux protein), COG0612 (predicted Zn-dependent peptidases), COG0006 (Xaa-Pro aminopeptidase), COG1686 (D-alanyl-D-alanine carboxypeptidase), and COG0024 (methionine aminopeptidase) among others. Similarly, in the genome of *Pseudomonas fluorescens* SRM1 isolated from spoiled milk, the operon containing proteases, lipases and the ABC-transporter, which directs enzyme secretion, was identified^45^. High enzymatic activity is required for efficient utilization of complex compounds from which bacteria produce energy. Furthermore, Gene Ontology system was used to determine the biological relevance of genes and gene products. In KM02, 870 GO terms were assigned to biological process, 77 GO terms to cellular component and 680 GO terms to molecular function (Supplementary Fig. S1). Among the ‘biological process’ GO terms, the most abundant were genes involved in ‘metabolic process’ (GO:0008152) and ‘cellular process’ (GO:0009987) as they consist of ancestor annotations of predicted genes, which are also annotated to child GO terms representing more specific entities^46^. Among the child terms that confirmed a wide spoilage potential of KM02, those containing: ‘catabolic’, ‘proteolysis’, ‘protein’, ‘lipid’, and ‘fatty acid’ phrases were revised and selected. In the ‘organic substance catabolic process’ GO term, the most abundant genes were genes involved in chemical reactions and pathways of organonitrogen compounds, organic acids, organic cyclic compounds, carbohydrates, organophosphates, macromolecules, proteins and lipids (Fig. 6A). In ‘cellular catabolic process’ group, which indicates the activity of individual cells, the most abundant pathways were pathways resulting in the degradation of aromatic compounds, nitrogen compounds, drugs, neurotransmitters, macromolecules, peptides, and sulfur compounds (Fig. 6B). As reported in Liu et al.^47^, the transcriptome of the *P. fluorescens* strain strongly associated with food spoilage differed from the RpoS-mutant strain in regard biological processes; the greatest differences were seen in GO biological processes such as signaling, protein catabolic process and secretion. Because *RpoS* contributes to the spoilage activities of *P. fluorescens*^48^, we can conclude that the abovementioned significantly downregulated genes are strongly involved in the spoilage of foods. Regarding the secretion system, according to the cellular component GO categories, the presence of the T2SS complex and transmembrane transporter activity molecular function was also noted. Among the other molecular function terms, the most abundant were ‘catalytic activity’, and those important for protein and lipid degradation were ‘hydrolase activity’, ‘catalytic activity, acting on a protein’, and ‘hydrolase activity, acting on ester bonds’ (Table 2). The functions of the assigned genes were also deduced on the basis of the sequence similarity of their presumptive protein products to the protein motifs in the Pfam database^49^. From among a wide range of annotated Pfam protein domains, only those related to hydrolase activity, protein secretion, lipid degradation and other fish spoilage aspects were selected, and their genome abundances are presented in Table 3. These data will significantly complement the current knowledge on the lipolytic activity of pseudomonads. Highly enriched Pfam domains were involved in the hydrolase activity represented by Aminohydro_1 and Abhydrolase_1 with relative abundances of 11,828 and 7768, respectively. Furthermore, most of the Pfam annotations assigned to the KM02 genome were related to peptidase activity, e.g., Peptidase_M20, Peptidase_M24 or Peptidase_M23. Most of these enzymes are classified as metallopeptidases, whose catalytic activity involves metals^50^. Proteins and lipids degraded to smaller molecules such as oligopeptides or single fatty acids are further metabolized by bacteria to form derivatives with undesirable odors. For example, ELFV_dehydrog represents the family of dehydrogenases of amino acids that catalyze the oxidative deamination of an amino acid to its keto acid analogs, known from spoiled fish^51^. In the context of lipid degradation, Pfam results revealed the presence of proteins representing Lipase_3 and Lipase_GDSL domains with abundance values of 2131 and 1246, respectively. Our results are in agreement with the work of Lo et al.^45^, who sequenced the *P. fluorescens* SRM1 strain and found that its genome contains heat-stable lipases encoded by *lipA* and *lipB* genes, which are responsible for spoilage of raw milk^45^.

**Table 2:** Selected GO terms of annotated *P. psychrophila* KM02 genome.

Biological domain	GO term	Definition according to QuickGO browser (https://www.ebi.ac.uk/QuickGO/)	Genome abundance
GO cellular component	1902494//catalytic complex	A protein complex which is capable of catalytic activity	23,765
	0098796//membrane protein complex	Any protein complex that is part of a membrane	20,030
	1902495//transmembrane transporter complex	A transmembrane protein complex which enables the transfer of a substance from one side of a membrane to the other	15,286
	0015627//type II protein secretion system complex	A large protein complex, containing 12–15 subunits, that spans the cell envelope of Gram-negative bacteria and mediates the movement of proteins into the extracellular environment	767
GO molecular function	0016787//hydrolase activity	Catalysis of the hydrolysis of various bonds, e.g. C–O, C–N, C–C, phosphoric anhydride bonds, etc	284,201
	0008233//peptidase activity	Catalysis of the hydrolysis of a peptide bond	39,142
	0022857//transmembrane transporter activity	Enables the transfer of a substance, usually a specific substance or a group of related substances, from one side of a membrane to the other	207,467
	0140096//catalytic activity, acting on a protein	Catalytic activity that acts to modify a protein	85,264
	0016788//hydrolase activity, acting on ester bonds	Catalysis of the hydrolysis of any ester bond	44,622

**Table 3:** Selected Pfam annotated domains in *P. psychrophila* KM02 genome.

Pfam ID/name	Description/putative function	Genome abundance
Amidohydro_1	A large metal dependent hydrolase superfamily	11,828
Abhydrolase_1	A superfamily of hydrolytic enzymes including proteases, lipases, peroxidases, esterases, epoxide hydrolases and dehalogenases	7768
AA_permease_2	Integral membrane proteins involved in the transport of amino acids into the cell	6643
Abhydrolase_6	Family contains alpha/beta hydrolase enzymes of diverse specificity	6099
MMPL	Putative integral membrane proteins from bacteria with probably function of lipid transport	5848
Peptidase_M20	Family includes a range of zinc metallopeptidases belonging to several families in the peptidase classification	5763
CN_hydrolase	Family contains hydrolases that break carbon–nitrogen bonds	5612
Amidase	A large group of hydrolytic enzymes that catalyse the hydrolysis of amide bonds (CO-NH2) of diverged substrates	5466
T2SSE	Family contains components of both the Type II (T2SS) and Type IV (T4SS) protein secretion system from Gram-negative bacteria	5142
Hydrolase_4	Domain found in bacteria and eukaryotes; the majority of the members in this family carry the exopeptidase active-site residues	4896
MotA_ExbB	Family groups together integral membrane proteins that appear to be involved translocation of proteins across a membrane	4393
Peptidase_M24	Family contains metallopeptidases that belong to MEROPS peptidase family M24	3955
M20_dimer	Domain consists of 4 beta strands and two alpha helices which make up the dimerisation surface of members of the M20 family of peptidases	3075
Secretin	Family includes: protein D that is involved in the general (type II) secretion pathway (GSP) within Gram-negative bacteria, a signal sequence-dependent process responsible for protein export	3050
Peptidase_M23	Members of this family are zinc metallopeptidases with a range of specificities	2758
Lon_C	The Lon serine proteases must hydrolyse ATP to degrade protein substrates; classified as family S16 in Merops	2446
Zn_protease	Family annotated as being ATP-dependant zinc proteases	2408
ELFV_dehydrog	Family that catalyze the oxidative deamination of an amino acid to its keto acid derivatives	2372
Peptidase_S11	Include a wide range of peptidase activity, including exopeptidase, endopeptidase, oligopeptidase and omega-peptidase activity	2249
Cys_Met_Meta_PP	Family includes enzymes involved in cysteine and methionine metabolism; acting as a coenzyme in a multitude of reactions, including decarboxylation, deamination and transamination	2191
Autotransporter	Family corresponds to the presumed integral membrane beta-barrel domain that transports the proteins products through the outer membrane	2160
Secretin_N	Domain found in bacterial type II/III secretory system proteins	2132
Lipase_3	A domain with an alpha/beta hydrolase fold that hydrolyse ester linkages of triglycerides	2131
Amidohydro_2	Amidohydrolases related to Amidohydro_1, family includes adenine deaminase that hydrolyses adenine to form hypoxanthine and ammonia	2066
Peptidase_M3	Group of metallopeptidases (oligopeptidases) that cleave medium sized peptides	1757
Ser_hydrolase	Family with serine hydrolase activity	1683
Peptidase_C13	Family of cysteine proteases that hydrolyses a peptide bond using the thiol group of a cysteine residue as a nucleophile	1623
Peptidase_S9	Family of serine-type peptidase activity	1622
Aminopep	Family of bacterial proteins has a conserved HEXXH motif, suggesting that members are putative peptidases of zincin fold	1550
Abhydrolase_2	Family consists of phospholipases and carboxylesterases with broad substrate specificity	1527
Lipase_GDSL	GDSL esterases and lipases are hydrolytic enzymes with multifunctional properties	1246
FA_desaturase	enzymes that catalyse the insertion of a double bond at the delta position of fatty acids	1240

### Effect of subMICs of BPEO, TEO, and their major compounds on *P. psychrophila* KM02 lipolytic activity

In light of the increasing use of EOs as modern fish biopreservatives, the current study assessed the anti-lipolytic potentials of BPEO and TEO and their major compounds toward KM02. Although the spoilage of fishery products is mainly caused by gram-negative microbes^51^ it is advisable to inhibit pseudomonad metabolic activity in seafoods. For this study, the subMIC concentrations of all agents were used; the concentrations of EOs above subMIC levels in foods can be sensorily unacceptable for consumers^52^. Many studies have demonstrated that plant EOs (e.g., oils of cinnamon and glove) can suppress bacterial metabolic activities/production of virulence factors when used at subMIC concentrations^53^. However, to date the inhibition of lipase production by plant- derived antimicrobials has only been shown in *Serratia marcescens* and *P. fluorescens* cultures^54,55^.

**Figure 7:**
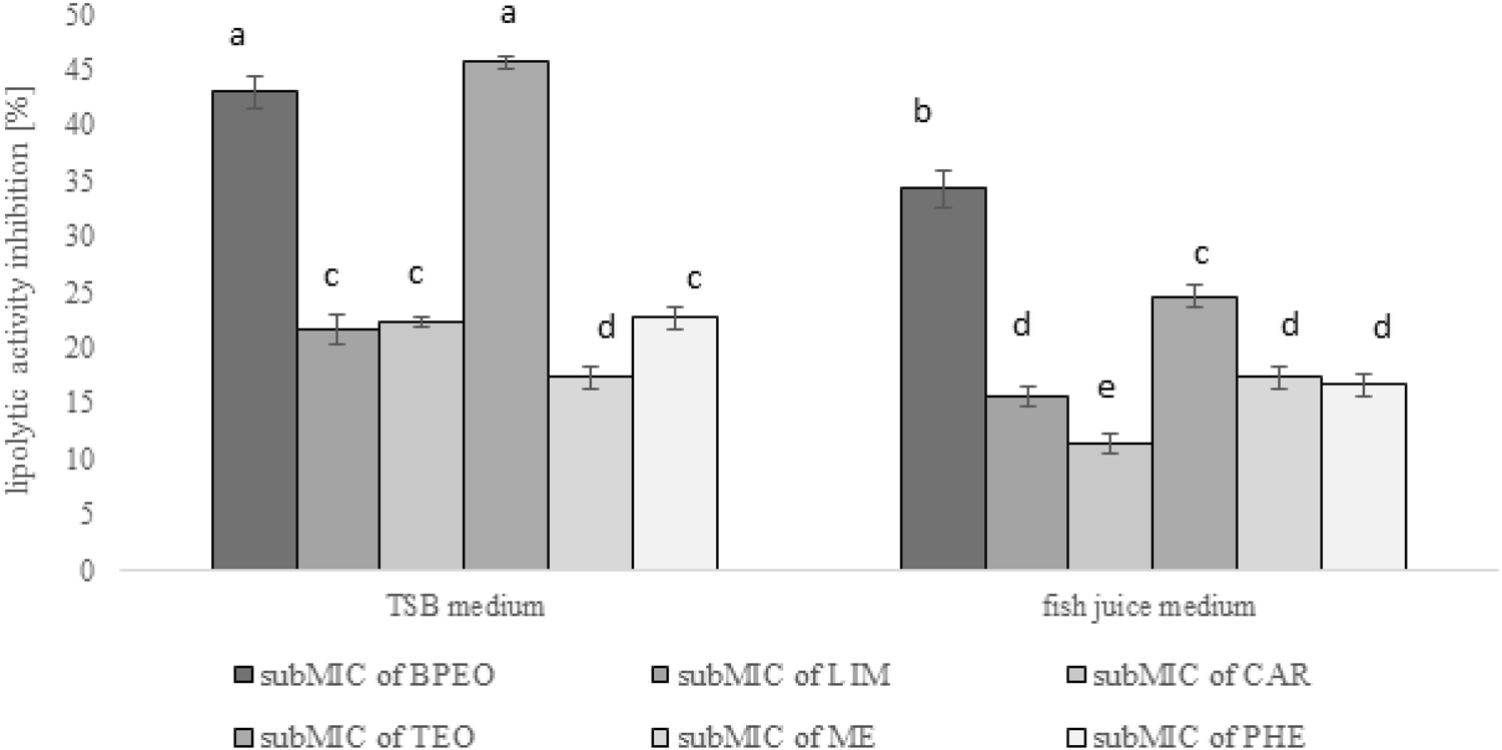
Inhibition of lipolytic activity of P. psychrophila KM02 grown on modified TSB and fish juice medium supplemented with subMICs of black pepper (BPEO) and tarragon essential oils (TEO), limonene (LIM), β-caryophyllene (CAR), methyl eugenol (ME), and β-phellandrene (PHE). Values are calculated from three independent replicates. Error bars represents standard deviation values. The same letter indicates not statistically differences in expression as provided by Tukey’s test after ANOVA analysis (F = 308.6, p < 0.05).

To investigate whether the analyzed compounds changed the lipolytic activity in KM02 cells, first, the spectrophotometric method with p-NPP reagent was used. As presented in Fig. 7, all treatments resulted in a considerable lipolysis decrease depending on the compound and medium, and it ranged from 11 to 46%. The highest inhibition potential was observed for the bulk of BPEO and TEO applied in modified TSB medium, and it was approximately twice that of major compounds used alone. The anti-lipolytic action of EO obtained from juniper toward fish-related *P. fluorescens* was also investigated, where the whole oil inhibited lipase production by 45%, while its major compounds, i.e., α-pinene and sabinene were significantly less effective^55^. A similar outcome was observed in the context of proteolytic enzyme inhibition in KM02, which was reduced to less than 20% by PHE and 28% by CAR, while TEO and BPEO resulted in significantly higher effects^8,14^. The explanation of such findings may be related to the lower antimicrobial effect of single terpenes, which was also seen in in vitro tests^52^. Notably, a combination of two different EO constituents or the presence of minor components in the entire EO volume can cause additive or synergistic antimicrobial effects^56^. For example, the inhibitory effect of LIM on *P. aeruginosa* was enhanced by the addition of on equal volume of eucalyptol^57^.

In fish juice medium, the overall anti-lipolytic activity was significantly (p < 0.05) lower than that under i*n vitro* conditions, with the exception of ME. However, based on the results, this compound inhibited lipolytic activity by only 17%. The decreased antimicrobial effectiveness of EOs and their major constituents is probably due to the more complex composition and physiochemical characteristics of the medium extracted from fish fillets. According to our previous study^55^, fish juice medium constituted 1.8 mg/g protein and 0.0635 mg/g lipids, which ideally mimics fish muscle conditions. These food components usually considerably reduce EOs bioactivity and protect bacteria by absorbing some volume of added EOs^58^. Therefore, to maintain equal antimicrobial efficacy in real food matrices where high molecular compounds are present, higher concentrations of antimicrobials are needed^59^. Furthermore, *P. psychrophila* was the least sensitive analyzed fish isolate for rosemary extract applied in a food model of common carp fillets, and its lipolytic potential was arrested by only one day in relative to the control culture^7^.

**Figure 8:**
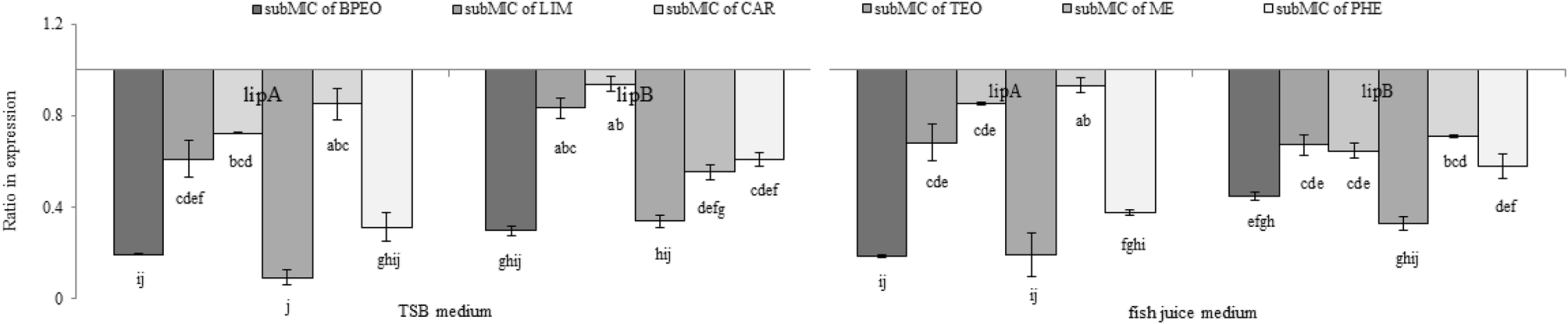
Ratio in expression of *lipA* and *lipB* genes in *P. psychrophila* KM02 grown on modified TSB and fish juice medium supplemented with subMICs of black pepper (BPEO) and tarragon essential oils (TEO), limonene (LIM), β-caryophyllene (CAR), methyl eugenol (ME), and β-phellandrene (PHE). Values are calculated from three independent replicates. Error bars represents standard deviation values. The same letter indicates not statistically differences in expression as provided by Tukey’s test after ANOVA analysis (F = 41.97, p < 0.05).

Similarly to *Actinobacter baumannii* and *Vibrio cholerae* strains, the genome of KM02 harbors genes encoding lipases (*lipA, lipB*)^60^. In this work, the anti-lipolytic activity of subMICs among the compounds was verified by RT-qPCR experiments and evaluation the expression of genes encoding lipases (*lipA, lipB*) in KM02 was evaluated. Based on the Pffafl calculations, the ratio of the expression of the *lipA* gene encoding the major lipase synthase ranged from 0.1 to 0.9 regardless of the culture medium used (Fig. 8). The highest decrease in *lipA* gene transcription was observed in cells treated with BPEO and TEO, which confirmed the phenotypic observations. Because the *lipA* and *lipB* genes are linked in a single operon, a disruption of even one of them results in a lipase-negative phenotype^61^. In the previous work, the *LipB* gene was also downregulated to the highest extent by whole EOs. According to the work of Christensen et al.^62^, the absence of the LipB protein in *Serratia proteamaculans* resulted in no spoilage of milk-based products. The aforementioned works indicate that changes in the expression of the *lipA* and *lipB* genes may result in a reduced rate of food biodeterioration^6^.

### Effect of subMICs of BPEO, TEO, and their major compounds on ***P. psychrophila*** KM02 T2SS

LipA and LipB are known type 2 substrates that degrade lipids^60^. Ogierman et al.^60^ noticed that a lipase-deficient *lipA* mutant of *Vibrio cholerae* was not able to grow on olive oil, and complementing the T2SS mutant with a plasmid expressing LipAB did not reverse this defect suggesting that secretion of lipases is T2SS-dependent. T2SS apparatus proteins are considered antimicrobial targets; thus in this work changes in the mRNA levels of T2 secretome genes in KM02 cells were evaluated. For RT–qPCR experiments, KM02 cells were treated with subMICs of BPEO, TEO and major compounds of the oils both in vitro and in fish juice medium mimicking the seafood ecosystem.

**Figure 9:**
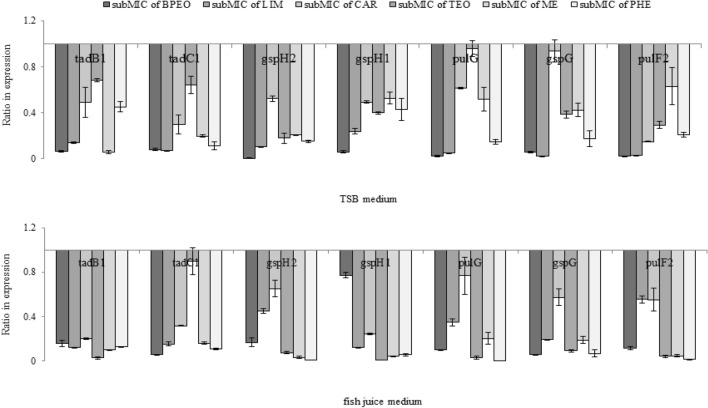
Ratio in expression of type II secretion system genes in *P. psychrophila* KM02 grown on modified TSB and fish juice medium supplemented with subMICs of black pepper (BPEO) and tarragon essential oils (TEO), limonene (LIM), β-caryophyllene (CAR), methyl eugenol (ME), and β-phellandrene (PHE). Values are calculated from three independent replicates. Error bars represents standard deviation values. The statistically differences in expression was provided by Tukey’s test after ANOVA analysis (F = 41.32, p < 0.05).

Based on bioinformatic analysis of the KM02 genome, the following 7 potential genes responsible for T2SS function were identified: *pulG* and *gspG* genes encoding pseudopilins PulG and GspG respectively; *tadB1* and *tadC1* genes encoding integral proteins of the inner membrane involved in the general secretion pathway (GSP); *gspH2* and *gspH1* genes encoding proteins required for energy-related secretion from the periplasm; and *pulF* gene involved in lipase export (Supplementary Table S3). As presented in Fig. 9, regardless of the agents used, the expression levels of most T2SS genes were inhibited to relative levels of between 0.9 and 0.02 that of control. Under in vitro conditions the most efficient treatments for downregulation of the expression of T2SS genes were BPEO and LIM, with relative transcript levels ranging from 0.2 to 0.02 that of control. In fish juice medium the highest reductions in the mRNA levels of T2SS genes were recorded for TEO and its singular components (i.e., ME and PHE). The most considerable inhibition concerned *gspH1* (0.05) and *gspH2* (0.02). These results are in line with the work of Jain, Nale and Dabur^63^, in which the response of pseudomonads to natural antimicrobials was evaluated at a proteomic level. Downregulation of proteins involved in secretion systems (e.g. xcp, PilS) in *P. aeruginosa* was caused by water extracts of the active fraction of catechins from *Saraca asoca* flowers^63^. Additionally, in the work of Singh et al.^64^, the authors noted the role of thyme EO in targeting the virulence arsenal regulated by the T2SS of *Xanthomonas oryzae* pv. *oryzae* strains. The downregulation of virulence gene expression in *Xanthomonas* strains remained insignificant when the bacteria were treated with thymol alone^64^.

### Changes in *P. psychrophila* KM02 growth in salmon-model products with subMICs of BPEO/TEO

The observed effect of BPEO and TEO on the T2SS-dependent lipolytic activity of the KM02 strain, triggered the need to verify the biopreservative properties of the examined agents in salmon-model products. The application of EOs in food requires additional techniques to mask their strong odor and simultaneously ensure their effectiveness^65^. In this work, an oil-vinegar marinade supplemented with subMICs of BPEO and TEO was formulated to improve the quality of fresh salmon fillets. The model product inoculated with standard amount of KM02 was next packed under vacuum conditions to prevent the degradation of EO components by oxygen.

**Figure 10:**
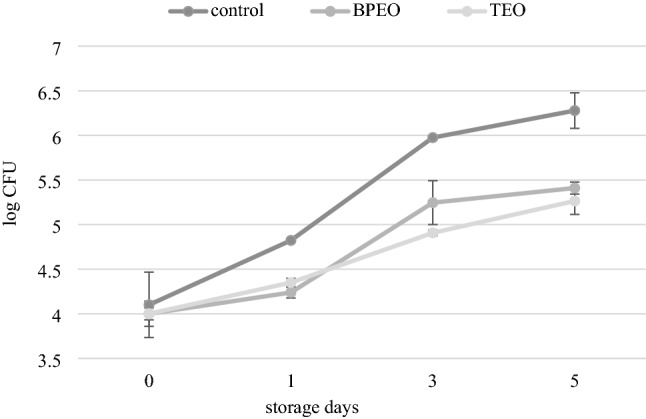
Combined effect of marinade and BPEO/TEO on *P. psychrophila* KM02 counts in salmon fillets stored at 4 °C in vacuum conditions. Data points represent the mean taken from two replicate experiments and error bars indicate the standard deviations (SD). The statistically differences was provided by Tukey’s test after ANOVA analysis (F = 50.34, p < 0.05).

The KM02 count after 1, 3, and 5 days of storage were evaluated (Fig. 10). After 1 day from an initial value of 4 log CFU/g, the KM02 control culture reached 4.8 log CFU/g, while BPEO and TEO impeded cell proliferation to 4.2 and 4.3 log CFU/g, respectively. At 3 and 5 days of storage, both treatments resulted in a significant (p < 0.05) reduction in KM02 counts in relation to the control product, where only marinade and vacuum packaging were applied. KM02 cells in the control sample reached a critical spoilage value of 6 log CFU/g between 3 and 5 days of refrigerated storage. BPEO- and TEO-treated samples did not exceed the value of 5.5 log CFU, which indicates the antimicrobial effect of marinade supplemented with EOs. Interestingly, even as strictly aerobic bacteria, KM02, was still able to proliferate under vacuum conditions. A substantial number of pseudomonad cells, despite vacuum packaging, were also observed in refrigerated trout fillet^66^. This is probably due to an inadequate barrier material used for packaging or not completely evacuating the gas from samples and the ability of cells to thrive in microaerophilic conditions. However, in comparison with aerobic storage, reducing the amount of oxygen results in a considerable decrease in microbiological counts and is an effective method for fish preservation based on hurdle technology^67^. Inhibitory effects of marinades enriched with oregano, rosemary and juniper EOs on the growth kinetics of psychotropic bacteria- contaminated foods were also observed by Siroli et al.^68^. In that work, marination showed the highest inhibition against *Pseudomonas* spp. and total coliforms. The molecular studies of Wu et al.^69^ revealed that selective compounds of EOs may competitively interact with the ATP binding site of the DNA gyrase B subunit of bacteria. Thus, natural antimicrobials combine with DNA to form a complex that eventually leads to DNA degradation, blocking cell transcription and replication^70^. Some bioactive agents may also cause the rearrangements of the nucleic acid double chain^21^. Interference with nucleic acids by bioactive compounds of EOs regulates bacterial metabolism and proliferation^21^. Moreover, aside from the ability to improve the safety and shelf-life of marinated fish, the utilization of EOs may also enhance consumers’ willingness to buy, in light of the recent increasing consumption of clean-label products^71^.

## Conclusions

In this work, bioinformatic analysis of genome and pangenome of KM02 revealed the ability of the strain to spoil foods with high content of proteins and lipids. Inactivation of T2SS by subMICs of BPEO and TEO resulted in reduced synthesis and secretion of lipases in KM02. The expression levels of most T2SS genes as well as *lipA* and *lipB* genes encoding lipases of the KM02 strain were downregulated by EOs. These features were observed in in vitro conditions and fish juice medium which mimicked the seafoods ecosystem. The biopreservative properties of BPEO and TEO were confirmed in salmon-model products; the oil-vinegar marinade supplemented with subMICs of the examined EOs impeded KM02 proliferation in comparison to the growth of bacteria in the control product, where only marinade and vacuum packaging were applied. Marinade supplemented with subMICs of BPEO and TEO can improve the quality of fresh salmon fillets in light of the recent increasing consumption of clean-label products.

## Supplementary Information

Supplementary Information.

## Data Availability

The data that support the findings of this study are available from the corresponding author upon reasonable request. All of the sequencing data were deposited in NCBI under Accession Number NZ_CP049044.1.
