# Supplementary material for: Black pepper and tarragon essential oils suppress the lipolytic potential and the type II secretion system of P. psychrophila KM02
Source: Sci Rep. 2022 Mar 31;12:5487. doi: 10.1038/s41598-022-09311-9 (PMC8971419; doi:10.1038/s41598-022-09311-9)
Supplement: Supplementary file 1 — Supplementary Information. [file 41598_2022_9311_MOESM1_ESM.pdf]

Supplementary data

Black pepper and tarragon essential oils suppress the lipolytic potential and the type II secretion system of *P. psychrophila* KM02

Natalia Tomaś [a]*, Kamila Myszka [a], Łukasz Wolko [b]

[a] Department of Biotechnology and Food Microbiology, Poznan University of Life Sciences, Wojska Polskiego 48, 60-637 Poznań, Poland

[b] Department of Biochemistry and Biotechnology, Poznan University of Life Sciences, Dojazd 11, 60-632, Poznań, Poland

* corresponding author: natalia.tomas@up.poznan.pl

Supplementary Table 1. The list of primers used in RT-qPCR experiments.

**Gene name**	**Forward primer**	**Reverse primer**	**Tm °C**	**Product length**
lipA	GTGGGGCAATTGGTTTGA	TGATTGACCATGCGCTGA	57	148
lipB	CTACCTTTTTGTTACCCGTT	ATCACGTCGTAGCATTTC	53	118
tadB1	CCCAGTACCAAAGCCGTCAT	CAACGTTCAGATGGGGGTGA	60	231
tadC1	TGCTGAAGAATCACGCAGGT	AGAGACGGCAACAGGAAGTG	60	262
gspH2	TCCACCAGCACTTGCAGATT	CCTCGCAACGTGGTTTTACC	60	244
gspH1	CGCTTTTGATGTCTGCCACC	CGCTACTTCAACAGCCTGGA	60	244
pulG	CCACTGACTGGGAAAGTCCG	AAGACCTGTTGCAGGACGAG	60	179
gspG	TTGGTGGTACTGGTGGTCCT	CTGCAAGCCTTGTTCGGTTG	60	177
pulF	TCAGCCAGGAACTGACAACC	CACAAGTGCAACGTAGAGCG	60	200

Supplementary Table S2. Features of *Pseudomonas psychrophila* KM02 genome.

**Attributes**	**Values**
Genome size	5,313,922 bp
GC content	57.4 %
Plasmid	0
Total predicted genes	4,813
Total CDS	4,713
Pseudogenes	54
Total RNAs	100
rRNAs (5S, 16S, 23S)	25 (9, 8, 8)
tRNAs	71
ncRNAs	4

Supplementary Table S3. Characteristic of the selected CDSs involved in the type II secretion system of *P. psychrophila* KM02.

CDS	Gene name	Gene definition and probable role
WP_048352188.1	*tadB1*	Type II secretion system F family protein, highly hydrophobic integral protein of the inner membrane involved in a general secretion pathway (GSP) for the export of proteins
WP_046810475.1	*tadC1*	Type II secretion system F family protein, highly hydrophobic integral protein of the inner membrane, involved in a general secretion pathway (GSP) for the export of proteins
WP_019825587.1	*gspH2*	Type II secretion system GspH family protein, required for the energy-dependent secretion of extracellular from the periplasm
WP_019825589.1	*gspH1*	Type II secretion system GspH family protein, required for the energy-dependent secretion of extracellular from the periplasm
WP_019825591.1	*pulG*	Type II secretion system protein, pseudopilin PulG, one of the secretion pseudopilins is found to assemble into pilus-like bundles
WP_046809061.1	*gspG*	Type II secretion system major pseudopilin GspG, it delivers toxins and a range of hydrolytic enzymes to the cell surface or extracellular space
WP_019825560.1	*pulF*	Type II secretion system F family protein involved in the export of proteins (toxins and a range of hydrolytic enzymes including proteases, lipases and carbohydrate-active enzymes) to the cell surface or extracellular space

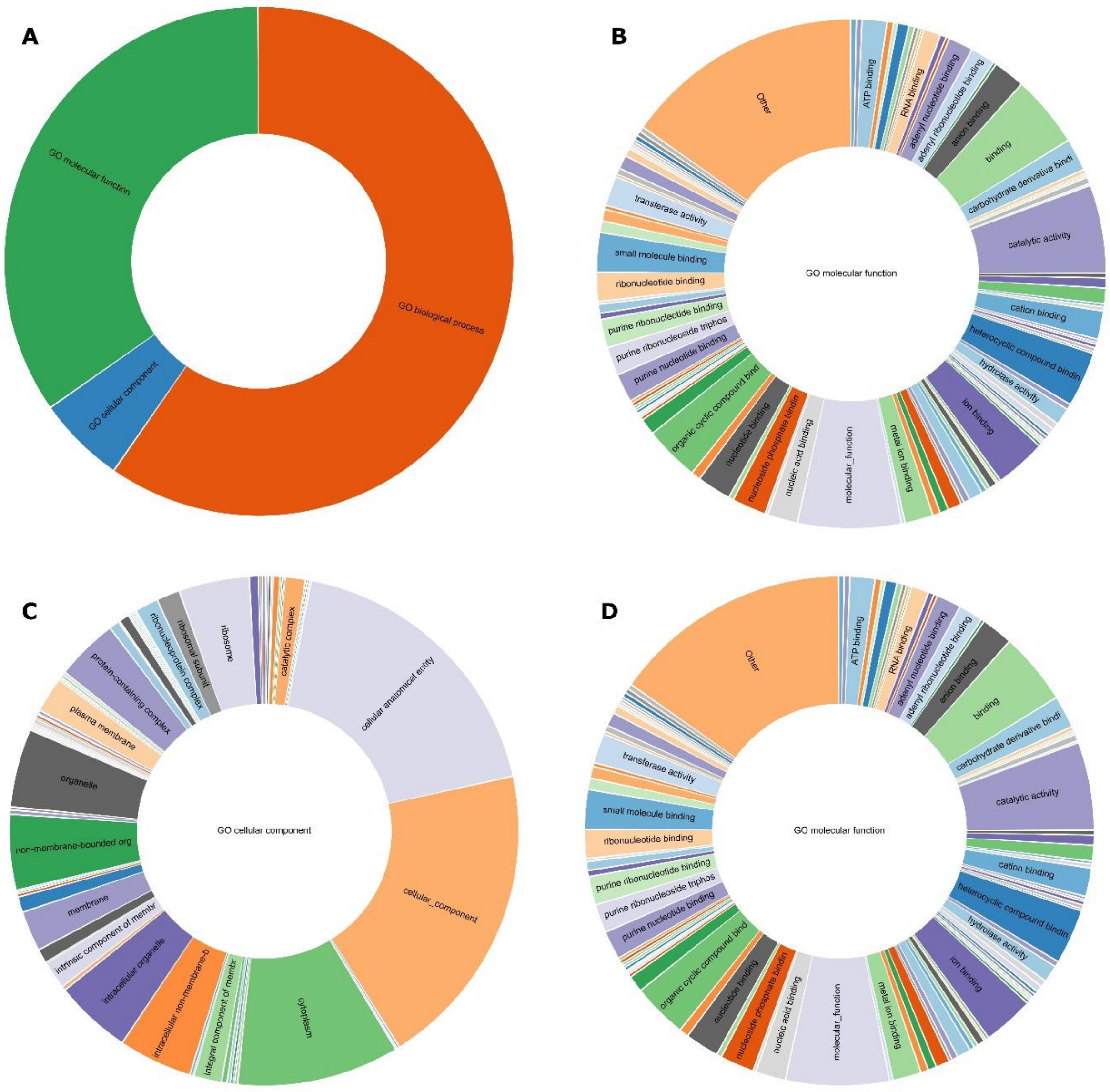

Supplementary Fig. S1 Distribution of GO annotations of *P. psychrophila* KM02 genome.

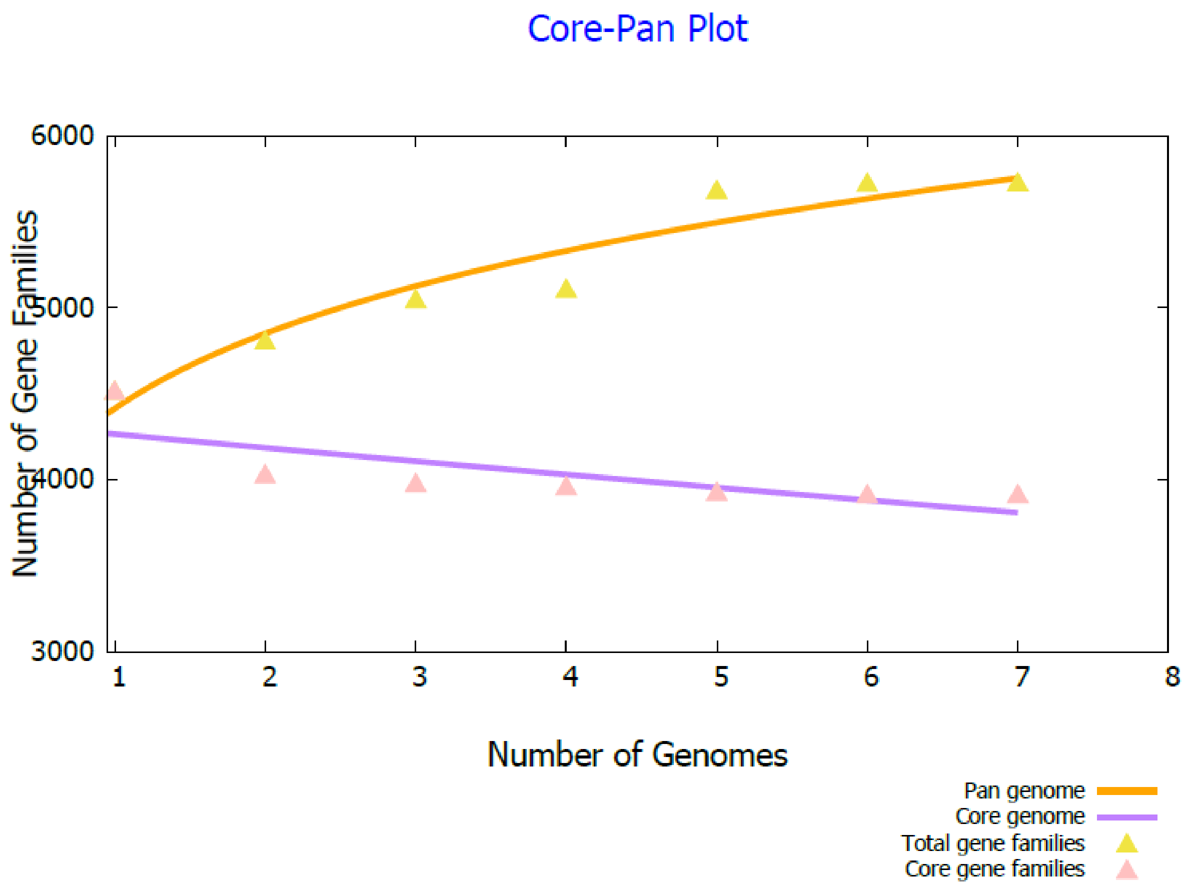

Supplementary Fig.S2. Core-pangenome plot of *P. psychrophila* species.
